# Supplementary material for: Clearing the Fog: A Scoping Literature Review on the Ethical Issues Surrounding Artificial Intelligence-Based Medical Devices
Source: J Pers Med. 2024 Apr 23;14(5):443. doi: 10.3390/jpm14050443 (PMC11121798; doi:10.3390/jpm14050443)
Supplement: Supplementary file 1 [file jpm-14-00443-s001.zip › jpm-2950358-supplementary.pdf]

Supplementary material

Supplementary Table S1. This table contains a summary of the relevant law/directives related to the main topics of ethical concerns about AI.

Ethical and Social issue	Field	Law/Directive
**Human Dignity and Integrity of user**	Human Rights	- Universal Declaration of Human Rights (United Nations);<br>- Convention for the Protection of Human Rights and Fundamental Freedoms (Council of Europe);<br>- European Charter for Fundamental Rights (European Union);<br>- Draft recommendation of the Council of Europe on the promotion of the human rights of older persons;<br>- European Charter of the Rights of Older People in need of long-term care and assistance;
**Privacy**	Data Protection	- Regulation (EU) 2016/679 of the European Parliament and of the Council of 27 April 2016 on the protection of natural persons with regard to the processing of personal data and on the free movement of such data, and repealing Directive 95/46/EC (General Data Protection Regulation);<br>- Directive (EU) 2016/680 of the European Parliament and of the Council of 27 April 2016 on the protection of natural persons with regard to the processing of personal data by competent authorities for the purposes of the prevention, investigation, detection or prosecution of criminal offences or the execution of criminal penalties, and on the free movement of such data, and repealing Council Framework Decision 2008/977/JHA;<br>- Directive 2006/24/EC of the European Parliament and of the Council of 15 March 2006 on the retention of data generated or processed in connection with the provision of publicly available electronic communication services or of public communications networks and amending Directive 2002/58/EC;<br>- Directive 2002/58/EC of the European Parliament and of the Council concerning the processing of personal data and the protection of privacy in the electronic communications sector;<br>- Regulation (EU) 2017/745 of the European Parliament and of the Council of 5 April 2017 on medical devices, amending Directive 2001/83/EC, Regulation (EC) 178/2002 and Regulation (EC) 1223/2009 and repealing Council Directives 90/385/EEC and 93/42/EEC;<br>- Regulation (EU) 2017/746 of the European Parliament and of the Council of 5 April 2017 on in vitro diagnostic medical devices and repealing Directive 98/79/EC and Commission Decision 2010/227/EU
**Bioethics and clinical trials**	Medical Research	- World Medical Association Declaration of Helsinki-Ethical Principles for Medical Research involving human subjects;<br>- Opinion on the processing of health data by Article 29 Data Protection Working Party;<br>- Universal Declaration on Bioethics and Human Rights;<br>- Directive 2001/20/EC on the approximation of the laws, regulations and administrative provisions of the Member States relating to the implementation of good clinical practice in the conduct of clinical trials on medicinal products for human use;<br>- Convention for the Protection of Human Rights and Dignity of the Human Being with regard to the Application of biology and Medicine: Convention on Human Rights and Biomedicine (and Guide for Research Ethics Committee Members);<br>- Charter for the Rights of Older People in Clinical Trials;<br>- Council Directive 90/385/EEC of 20 June 1990 on the approximation of the laws of the Member States relating to Active Implantable Medical Devices as amended by Directive 2007/47/EC of 5 September 2007;<br>- Council Directive 93/42/EEC of 14 June 1993 concerning Medical Devices as amended by Directive 2007/47/EC of 5 September 2007;<br>- Directive 98/79/EC on In Vitro Diagnostic Medical Devices as amended by Directive 2007/47/EC of 5 September 2007;<br>- Commission Regulation (EU) 207/2012 of 9 March 2012 on electronic instructions for use of medical devices;<br>- Directive 2001/83/EC of the European Parliament and of the Council of 6 November 2001 on the Community code relating to medicinal products of human use

**New Technologies**	Liability and Safety	- Directive 85/374/EC on liability for defective products as amended by Directive 1999/34/EC;<br>- Directive 2011/24/EU on the application of patients' rights in cross-border healthcare;<br>- Directive 90/385/EEC on active implantable medical devices and Directive 93/42/EEC on medical devices and Directive 98/79/EC on in vitro diagnostic medical devices;<br>- RoHS Directive 2002/95/EC of the European Parliament and of the Council of 27 January 2003 on the restriction of the use of certain hazardous substances in electrical and electronic equipment;<br>- Directive 98/34/EC of the European Parliament and of the Council of 20 July 1998 amended by Directive 98/34/EC laying down a procedure for the provision of information in the field of technical standards and regulation and of rules on information society services<br>- OECD. OECD Legal Instruments. 2019 1 February 2023]; Available from: https://legalinstruments.oecd.org/en/instruments/OECD-LEGAL-0449.<br>- Google. Our Principles – Google AI. 2019 01/02/2023]; Available from: https://ai.google/principles/.<br>- ACM. SIGAI - Artificial Intelligence. 2022; Available from: https://www.acm.org/special-interest-groups/sigs/sigai.<br>- EU AI Act: https://www.europarl.europa.eu/topics/en/article/20230601STO93804/eu-ai-act-first-regulation-on-artificial-intelligence<br>- High-Level Expert Group on AI (AI HLEG), Ethics Guidelines for Trustworthy AI. 2018: https://digital-strategy.ec.europa.eu/en/library/ethics-guidelines-trustworthy-ai<br>- Id., Assessment List for Trustworthy Artificial Intelligence (ALTAI) for self-assessment | Shaping Europe's digital future. 2020: https://digital-strategy.ec.europa.eu/en/library/assessment-list-trustworthy-artificial-intelligence-altai-self-assessment<br>- Id., White Paper on Artificial Intelligence A European approach to excellence and trust. 2020: https://commission.europa.eu/publications/white-paper-artificial-intelligence-european-approach-excellence-and-trust_en<br>- UNESCO, UNESCO's Input in reply to the OHCHR report on the Human Rights Council Resolution 47/23 entitled "New and emerging digital technologies and human rights". 2021.

Supplementary Table S2. Summary of study technological and medical contexts and outcomes.

Context	Study	Main ethical issues raised	Key findings
**General**	McLennan et al. (2022) [42]	Accountability<br>Algorithmic bias	Embedded ethics is the most effective and easily implemented approach to solve ethical issues raised in medical technology, but a clear standard of practice is required
	Svensson et al. (2022) [43]	Autonomy<br>Algorithmic bias<br>Confidentiality<br>Informed consent	The concepts of autonomy and justice (with respect to the uniqueness and value of humanity) should be the priority for moral frameworks to develop improved medical technology regulations
	Martinho et al. (2021) [44]	Confidentiality<br>Fairness<br>Transparency	The perspectives of clinicians and a multidisciplinary approach are important for the design, development and implementation of AI in healthcare
	Donia et al. (2021) [45]	Algorithmic bias<br>Fairness	Solutions to the pitfalls of co-design for AI may include: design humility, reconceptualising representation and clarifying commitments to the values used for design
	Arima et al. (2021) [46]	Accountability<br>Autonomy<br>Confidentiality<br>Transparency	Compliance with universal requirements using integrated framework is recommended for efficacy and convergence
	Racine et al. (2019) [47]	Algorithmic bias<br>Confidentiality<br>Informed consent<br>Transparency	These ethical issues can be tackled by collaboration, improved training and awareness, adjustments to institutional codes of ethics and a dedicated institutional response
	Guan et al. (2019) [48]	Autonomy<br>Fairness	Specific guidelines, specifically for frontier AI fields, are needed to govern trustworthy AI in healthcare and medicine
	Quinn et al. (2021) [49]	Autonomy<br>Transparency	Strategy and governance are required to use AI effectively and this can be achieved by creating expert groups for development, verification and operation of medical technology

Arnold (2021) [50]	Algorithmic bias<br>Autonomy<br>Trust	It is essential clinicians engage with discussion and development of medical AI, in order to protect autonomy
Karmakar (2021) [51]	Transparency	There is a need for improvements to the remit of AI in healthcare, aligning discourse on legal and ethical frameworks and dismiss logical fallacies on the potential of AI
Montemayor et al. (2021) [52]	Transparency	Empathetic AI is impossible or unethical, it may also reduce the meaning and expectation of real human empathy, so therefore, human monitoring and emotional intervention is still necessary
Adlakha et al. (2020) [53]	Confidentiality<br>Informed consent	Data security and privacy are currently the biggest limitations of AI in healthcare, so building a strong security system to correct loopholes is essential
Ho (2019) [54]	Algorithmic bias<br>Confidentiality<br>Fairness<br>Transparency<br>Trust	Proactivity and vigilance are important in ensuring patient care is equitable using AI technology. A bioethical feedback loop can help provide anticipatory and ongoing guidance
Whitby (2015) [55]	Accountability	There is an urgent need for progress in machine medical ethics due to rapid development; Training for healthcare staff is required as well as a transition towards a no blame model for investigating incidents
Buruk et al. (2020) [56]	Accountability<br>Autonomy<br>Beneficence<br>Fairness<br>Transparency	There is a need to revise guidelines for AI health technologies supported by expert feedback, as currently these do not address prospective ethical issues
de Miguel et al. (2020) [57]	Algorithmic bias<br>Informed consent<br>Transparency	There is an urgent need to further develop regulatory framework regarding medical AI, as well as to develop new roles such as 'Health Information Counsellors'
Johnson (2020) [58]	Confidentiality<br>Fairness<br>Transparency<br>Trust	Best practice principles should be applied to AI and machine learning patient data in healthcare systems
Pasricha (2023) [59]	Algorithmic bias<br>Transparency<br>Privacy	Ethics training should be systematically integrated into medical device design programs and frameworks for ethical analysis are needed for entire device lifecycles
Reddy (2023) [60]	Algorithmic bias<br>Confidentiality<br>Transparency<br>Trust	Use of AI in healthcare requires bespoke and precise regulation
Zhang and Zhang (2023) [61]	Transparency<br>Trust<br>Confidentiality<br>Autonomy	Priority should be placed on systems underlying AI, such as improving data quality, management and sharing. Regulation is needed to ensure transparency and traceability, to satisfy multiple stakeholders throughout device lifecycle
Pruski (2023) [62]	Algorithmic bias<br>Trust	Prediction models present a unique challenge, as performance is likely to change in time (as populations change). There is a need for a monitoring and updating system to handle these models
Schicktanz et al. (2023) [63]	Accountability<br>Trust	Ai simulation can assist ethical reflections during the design stage of development
Adams (2023) [64]	Autonomy<br>Transparency	Explicability should be included as a new principles of bioethics
Love (2023) [65]	Autonomy	There are limitations of AI in medicine due to conflicts with Christian concepts of autonomy and hope
Couture (2023) [66]	Confidentiality<br>Fairness<br>Trust	Use of AI in population health presents unique risks relating to privacy, bias, and exacerbation of social inequality
Aquino (2023) [67]	Trust	More participation and increased diversity and inclusion in research are needed to avoid exacerbating health inequalities

	Chikhaoui et al. (2022) [68]	Fairness<br>Fairness<br>Trust	Increased education relating to AI is needed, to ensure individuals have autonomy over data usage and ability to withdraw consent
	Cobianchu et al. 2022 [69]	Algorithmic bias<br>Autonomy<br>Transparency<br>Algorithmic bias<br>Confidentiality	Perspective should always be patient centered, multidisciplinary development and involvement of stakeholders can support this
	De Togni et al. (2022) [70]	Fairness<br>Accountability<br>Algorithmic bias<br>Transparency	Medical AI should not replace but rather assist humans
	Iqbal et al. (2022)[71]	Trust<br>Fairness<br>Accountability<br>Autonomy	Adequate regulation for digital twins is needed as a matter of urgency
	Lewanowicz et al. (2022) [72]	Privacy<br>Confidentiality<br>Trust	Scientific research and validation is key to overcoming ethical concerns around privacy
	Martín-Peña (2022) [73]	Trust<br>Fairness	The field of Machine Ethics should be developed with a multi-theoretical approach to integrate values across disciplines and frameworks
	Elsa Papadopoulou (2022) [74]	Accountability<br>Accountability<br>Autonomy	Ethics, legal accountability and trustworthiness need to be embedded into design of AI systems for healthcare
	Pasricha (2022) [75]	Confidentiality<br>Algorithmic bias<br>Transparency	AI in medicine creates unique and emerging ethical challenges, education, policy development and lifecycle analysis are needed
	Refolo et al. (2022) [76]	Confidentiality<br>Autonomy<br>Confidentiality<br>Trust	Ethical models can be applied to analyzing digital therapeutics which incorporate AI
	Smallman (2022) [77]	Transparency<br>Accountability<br>Trust	A multiscale ethical framework can provide structure to address wide-reaching ethical implications of AI health technologies
**Decision support systems**	de Boer et al. (2021) [78]	Accountability<br>Algorithmic bias<br>Human bias<br>Transparency	Concerns of machine learning in healthcare (epistemic, existential, and legal) can be identified using technomoral change and technological mediation theory
	Braun et al. (2021) [79]	Accountability Autonomy<br>Transparency<br>Trust	'Meaningful human control' can be used as framework to discuss and solve the ethical issues of clinical decision support systems, although clinician-patient discussions are still essential when uncertainties are faced
	Rogers et al. (2021) [80]	Accountability<br>Algorithmic bias<br>Confidentiality<br>Fairness<br>Informed consent<br>Transparency<br>Trust	There is a need for further ethical evaluation, benchmarks, and security across the AI life-cycle, beyond that of the current level of ethical guidance
	Lysaght et al. (2019) [81]	Accountability<br>Algorithmic bias	At the patient level accountability is important, at the societal level justice and harm must be balanced, and transparency is important to ensure trust throughout. Deliberative framework can be drawn on for design and implementation

		Fairness<br>Integrity<br>Transparency	
	Astromske et al. (2021) [82]	Informed consent<br>Transparency<br>Trust	Currently robots are not held to performance standards (as these do not exist), therefore legislation is the only viable option to enforce standards
	Fletcher et al. (2021) [83]	Algorithmic bias<br>Fairness	Three basic criteria (appropriateness, fairness, and bias) can be used to evaluate the use of AI medical devices
	Nabi (2018) [84]	Algorithmic bias<br>Autonomy<br>Confidentiality	AI can be used to tackle inequity however input from medical, technological and policy experts are required. In the short term anticipatory and contextual design may be used, but in the long term national policy changes are needed
	Amann et al. (2020) [85]	Algorithmic bias<br>Autonomy<br>Beneficence<br>Fairness<br>Informed consent<br>Non-maleficence<br>Transparency	A lack of transparency (specifically 'explainability') in clinical decision support systems poses a threat to ethical values healthcare. Informed consent, the approval of medical devices, and liability are key points from the legal perspective
	Chen et al. (2023) [86]	Transparency<br>Trust	Efforts should be focused on explainability and transparency by design as well as clear definition of where responsibility for AI systems lies
	Hallowell et al. (2023) [87]	Autonomy<br>Trust<br>Algorithmic bias	Perceptions among many stakeholders is that AI offers potential for faster more accurate diagnosis. Concerns remain around algorithmic reliability and bias. Decision support based on AI should not replace human decision makers
	Lorenzini et al. (2023) [88]	Autonomy<br>Accountability<br>Transparency	AI tools for decision making presents implications for doctors and patients autonomy, if AI is 'overused' there is a risk patient loses ownership of decision making
	Cagliero et al. (2023) [89]	Algorithmic bias<br>Trust<br>Transparency<br>Fairness<br>Informed consent	There are incongruencies between different stakeholders' concerns and priorities. End user inclusion in development is key to success and uptake
	Redrup Hill et al. (2023) [90]	Trust<br>Confidentiality<br>Transparency	Clear regulations and guidelines are needed to manage the ethics relating to the influence of AI on human involvement on healthcare
	Ferrario (2023) [91]	Algorithmic bias<br>Transparency	Interdisciplinary strategies are needed to resolve challenges in developing AI systems
	Lorenzini et al. (2023) [92]	Informed consent<br>Autonomy<br>Transparency	Ethical and practical considerations need to be bridged to ensure informed consent is not compromised for medical AI
	Sharova et al. (2021) [93]	Confidentiality<br>Trust<br>Accountability	Standardization of ethical regulation can ensure trust and safety of AI technologies in healthcare
	Wellnhofer (2022) [94]	Confidentiality<br>Trust<br>Informed consent<br>Transparency	Current regulatory frameworks and guides for AI in medical devices do not cover all ethical and legal concerns. A risk-based regulatory approach is reccomeneded, including surveillance and clinical evaluation
**Big data**	Ballantyne et al. (2019) [95]	Fairness<br>Transparency	Deliberative framework can be used to identify relevant values and interested at stake for public-private partnerships in biomedical big data
	Howe et al. (2020) [96]	Algorithmic bias<br>Confidentiality	Big data has huge potential but also carries a risk of harm; The risks of this research must be accounted for and solutions should be sought on a societal and inter-personal level

	De Angelis et al. (2023) [97]<br>Liu and Wu (2023) [98]	Informed consent<br>Accountability<br>Algorithmic bias<br>Fairness<br>Confidentiality	Policy should be developed with multidisciplinary teams and awareness of ethical concerns should be raised<br>Federated learning and differential privacy are techniques which can be deployed to preserve privacy
**Robotics**	Fiske et al. (2019) [99]	Autonomy<br>Confidentiality<br>Fairness<br>Non-maleficence<br>Transparency	There is a need for further research into the ethical issues and broader societal concerns of AI technology in mental health care, as well as clear guidance, improved training and current service provision
	Steil et al. (2019) [100]	Accountability<br>Autonomy<br>Transparency	Further consideration of the roles and responsibilities of health care professional and robots in operating theatres, plus inter- and multidisciplinary collaboration and involvement of the public in this debate
	De Togni et al. (2021) [101]	Accountability<br>Algorithmic bias<br>Fairness<br>Transparency	AI can be used across different dimensions of 'intelligence' and this rematerialises the boundaries of human and machine identities. There is a need for developmental of conceptual, normative and ethical tools to evaluate AI technology
	Weber (2018) [102]	Confidentiality<br>Trust	Due to the development of sophisticated robots (particularly social and decision making agents) there is a need to restructure ethical, legal, and regulatory frameworks
	Bendel (2015) [103]<br>Shuaib et al. (2020) [104]<br>Boch et al. (2023) [105]	Accountability<br>Fairness<br>Confidentiality<br>Trust<br>Autonomy<br>Trust<br>Confidentiality<br>Transparency	Further research is required to solve problems in machine ethics, specifically with unput from patients and healthcare professionals<br>There is a need to better understand the challenges that technology brings to healthcare and core principles of humanity and patient-centred care should guide these devices<br>Sector specific ethical discussion and quantifiable characteristics to evaluate adherence to ethical principles are needed
**Rehabilitation**	Lanne et al. (2021) [107]	Autonomy<br>Confidentiality<br>Trust<br>Transparency	AI rehabilitation therapy brings both opportunities and challenges, however following guidance can lead to ethical implementation
**Medical education**	Leimanis et al. (2021) [108]	Algorithmic bias	AI technology is not yet ready to be the primary decision maker in healthcare but can be used as a digital assistant for healthcare professionals. Developers should adopt self-imposed ethical guidelines to reduce risk
**Monitoring technology for the elderly**	Ho (2020) [109]	Autonomy<br>Confidentiality	AI monitoring technology can be used to help facilitate older people to live independently however clinical and ethical factors must be considered, and older adults should be involved the design and research of this technology
**Mental health**	Luxton (2014) [110]	Accountability<br>Confidentiality<br>Transparency<br>Trust	The ethical and moral aspects of AI care providers must be used to guide the development of these systems
**Radiation technology**	Smith et al. (2019) [111]	Accountability<br>Algorithmic bias<br>Confidentiality<br>Transparency	It is essential for radiation clinicians to understand the risks and benefits associated with AI technology in this area, and a transparency, replicability, ethics, and effectiveness (TREE) life-cycle approach can be used as a tool for researchers and policy makers

**Chatbots**	Parviainen et al. (2022) [112]	Accountability<br>Trust	Chatbots affect decision making in clinical practice (via automation and rationality), and new ethical-political approaches and policy are required to implement chatbots safely
**Health apps**	Kuhler et al. (2022) [113]	Algorithmic bias<br>Autonomy<br>Confidentiality<br>Informed consent<br>Transparency<br>Trust	The ethical issues raised by paternalism within AI-healthcare apps must be debated regarding design and development
**Healthcare in low- and middle-income countries**	Kerasidou et al. (2021) [114]	Algorithmic bias<br>Fairness<br>Transparency<br>Trust<br>Value	Fair and appropriate AI requires successful development and implementation of national and international rules and regulations
**Adaptive AI**	Hatherley (2023) [106]	Informed consent<br>Fairness<br>Accountability	The evolving nature of adaptive AI systems presents unique challenges to health equity and quality of care. These are novel challenges and support is needed for monitoring and evaluation of entire lifecycles

Supplementary Table S3. Percentage of studies discussing different medical contexts (n=41). Certain studies addressed more than one medical context.

**Medical context**	**Number of studies (%)**
General	46 (59)
Surgery	3 (4)
Psychiatry	3 (4)
Dementia	1 (1)
Geriatrics	1 (1)
Occupational therapy	1 (1)
Nephrology	1 (1)
Medical education	7 (9)
Palliative care	1 (1)
Pathology	3 (4)
Gynaecology	1 (1)
Respiratory	1 (1)
Ophthalmology	2 (3)
Public health	7 (9)
